# Comparison of Predictive Value of Cardiometabolic Indices for Subclinical Atherosclerosis in Chinese Adults

**DOI:** 10.1371/journal.pone.0093538

**Published:** 2014-04-01

**Authors:** Ying Xu, Fang-fang Zeng, Li-ping He, Wen-hua Ling, Wei-qing Chen, Yu-ming Chen

**Affiliations:** 1 Guangdong Provincial Key Laboratory of Food, School of Public Health, Sun Yat-sen University, Guangzhou, People's Republic of China; 2 Department of Epidemiology and Biostatistics, School of Public Health, Guangdong Pharmaceutical University, Guangzhou, People's Republic of China; 3 Guangzhou Panyu Central Hospital, Guangzhou, People's Republic of China; University of Catanzaro Magna Graecia, Italy

## Abstract

**Objectives:**

Metabolic disturbances are well-known risk factors for atherosclerosis, but it remains unclear which cardiometabolic components are the predominant determinants. This study aimed to compare and identify the key determinants of carotid atherosclerosis in asymptomatic middle-aged and elderly Chinese.

**Methods:**

A community-based cross-sectional study including 3,162 apparently healthy residents aged 37–75 years was performed from July 2008 to June 2010 in Guangzhou, China. Carotid artery intima-media thickness (IMT) was assessed by B-mode ultrasound, and increased IMT was defined as IMT>1.00 mm. Obesity indices, blood pressure, fasting blood lipids, glucose and uric acid levels were determined. Principal components factor analysis was used to extract common factors underlying 11 metabolic factors.

**Results:**

Four common factors, defined as “adiposity,” “blood lipids,” “triglycerides/uric acid (TG/UA)” (in men) or “triglycerides/uric acid/glucose (TG/UA/Glu)” (in women), and “blood pressure,” were retained for both sexes. After adjustment for potential covariates, the “adiposity” factor showed the strongest positive association with increased IMT in men. Comparing the extreme quartiles, ORs (95% CI) of increased IMT were 4.64 (2.04–10.59) at the CCA and 2.37 (1.54–3.64) at the BIF), followed by “blood pressure”, the corresponding OR (95% CI) was 2.85 (1.37–5.90) at the CCA. Whereas, the four common factors showed comparable and weak relationship with increased IMTs, the ORs for quartile 4 vs. quartile 1 varied from 0.89 to 3.59 in women.

**Conclusions:**

Among the metabolic factors, “adiposity” and “blood pressure” play predominant roles in the presence of carotid atherosclerosis in men, but no key factor is identified in women.

## Introduction

Numerous epidemiologic studies have shown that metabolic disturbances play a key role in the development of cardiovascular disease (CVD). It is well established that metabolic factors, such as hypertension, dyslipidemia, obesity, impaired blood glucose and hyperuricemia, are associated with CVD, together accounting for over 90% of the population attributable risks [1]. Although all of these factors are important, it remains unclear which of them plays a predominant role in the development of CVD, and the predictive ability of each factor might vary between different populations. Previous studies have compared the ability of these metabolic factors to predict CVD risk, and the results were inconsistent. Various studies have identified increased blood pressure [2]–[6], high serum cholesterol [7]–[9], low HDL cholesterol [10], elevated fasting glucose [11], [12], and obesity [13] as strong predictors of CVD, but others have reported that these factors are similar in predictive power [14]–[16]. In addition, the predominant predictors might differ between men and women [17]–[19]. However, the apparent variations might be a result of differences in study design, data analysis, information obtained, and the confounding variables adjusted for. Moreover, current evidence on this issue is mainly derived from studies conducted in Western populations and seldom in Chinese, who have significantly different risk factor profiles [20]. In China, 32% of all deaths in 2005 were attributed to CVD [20], and by 2030, this statistic is expected to increase by >50% [21]. Further studies are needed to identify the key determinants of CVDs in the Chinese population.

Because atherosclerosis is the primary pathological manifestation of CVD before the onset of CVD events, effective prevention of atherosclerosis and inhibition of its progress will be key measures to the prevention and control of CVD. Thus, identification of the key determinants of atherosclerosis is fundamental to the establishment of a strategy for preventing atherosclerosis. In the study described herein, we selected 11 metabolic risk factors and compared their importance for the presence of subclinical atherosclerosis to find the most important determinant(s) or predictors(s) in middle-aged and elderly Chinese. Considering the high degree of inter-correlation among these metabolic risk factors, we used principal components factor analysis (PCFA) to derive common factors.

## Methods

### 1. Study population

From July 2008 to June 2010, we conducted a community-based cross-sectional study in urban Guangzhou, China. Participants were recruited from communities through community advertisement, by telephone, and by referral. Eligible individuals were apparently healthy residents aged 40–75 years who had lived in Guangzhou for at least 5 years. Individuals were excluded if they reported having previously confirmed diabetes, CVD, dyslipidemia or cancer; were taking any medication known to affect lipids or glycometabolism; or if they were suffering from a hearing disorder or mental disorder. A total of 3,162 individuals met the criteria and were interviewed. The study was approved by the Medical Ethical Committee of the Sun Yat-sen University and written informed consent was obtained from all participants.

### 2. Data collection

Participants' demographic and health-related characteristics (e.g., age, sex, education), health-related lifestyle (e.g., smoking status, drinking status, dietary habits, and physical activity), personal and family history of chronic disease, and use of medications were collected by means of face-to-face interviews conducted by trained staffs who used a structured questionnaire. Smoking status included non-smokers and smokers. A ‘smoker’ was defined by having smoked greater than 5 packages (100 cigarettes) during their lifetime and reporting smoked at the time of the interview. Drinking status included non-drinkers and drinkers. A ‘drinker’ was defined by having had regularly drink alcohol at least once a week for six consecutive months and reporting drunk at the time of the interview [22].

#### Anthropometric and laboratory measurements

Height and body weight were measured with the participant barefoot and dressed in light clothing. Body mass index (BMI) was calculated as weight (in kilograms) divided by height (in meters) squared. Waist circumference (WC) was measured with an elastic tape after exhalation, hip circumference was measured at the level of the pubic symphysis around the widest portion of the buttocks, and waist-hip ratio (WHR) was calculated as WC divided by hip circumference.

After a rest of ≥10 min, supine blood pressure was measured at least twice on the left arm with a mercury sphygmomanometer; a third measurement was taken on any occasion when the first two DBP or SBP values differed by more than 3 mmHg or 4 mmHg. At least two measurements were averaged for further analysis.

Venous blood samples were obtained after an overnight fast. The serum was separated within 2 hours and stored at −80°C. Serum total cholesterol (TC), high density lipoprotein-cholesterol (HDL-C), low density lipoprotein-cholesterol (LDL-C), total triglycerides (TG), fasting glucose (Glu), and uric acid (UA) were measured with a Hitachi 7600-010 automatic analyzer. The coefficient of variation for lipid measurements was 2.17% (at 5.03 mmol/L) for TC, 3.47% (at 1.70 mmol/L) for HDL-C, 4.67% (at 2.65 mmol/L) for LDL-C, 2.86% (at 1.14 mmol/L) for TG, 2.52% (at 4.45 mmol/L) for Glu, and 5.46% (at 303.5 mmol/L) for UA.

#### Carotid IMT measurements

The intimal-to-medial arterial wall thickness was measured bilaterally at the far wall of the carotid arteries with a high-resolution, 7.5 MHz linear-array transducer system (Aplio TOSHIBA, Japan) according to previously published protocols [23]. Briefly, the participants were examined in a supine position with the neck rotated in the direction opposite the probe. Three carotid segments on each side were measured at the peak of the R wave on a simultaneous electrocardiogram tracing: the distal common carotid artery (CCA) segment (1 cm proximal to the carotid bulb), the carotid artery bifurcation (BIF) (1 cm proximal to the flow divider), and the proximal internal carotid artery (ICA) segment (1-cm section of the internal carotid artery immediately distal to the flow divider). Inter-scan reproducibility for IMT was 0.89 with this software; the inter-reader and intra-reader reproducibilities were 0.98 and 0.86 for CCA-IMT, 0.72 and 0.68 for BIF-IMT, and 0.68 and 0.60 for ICA-IMT, respectively. Carotid atherosclerosis was defined as IMT≥1.00 mm [24].

### 3. Statistical analysis

Statistical analyses were performed separately for men and women. Descriptive statistics are presented as mean (standard deviation, SD) for continuous variables and count (%) for categorical variables. The differences between men and women were analyzed by the Student *t*-test or Mann-Whitney U test for continuous variables and the chi-square test for categorical variables.

As the primary objective of this study was to identify the nature of the factors underlying 11 relevant risk factors variables (BMI, WC, WHR, TC, HDL-C, LDL-C, TG, DBP, SBP, Glu, and UA), the data were subjected to factor analysis. The extraction method used was principal component analysis (PCFA) which extracts the observable variables. Factor analysis was used to uncover the latent structure of a set of variables. It reduces attribute space from a large number of variables to a smaller number of factors. The varimax rotation method which seeks to maximize the variances of the squared normalized factor loadings across variables for each factor was used to maintain uncorrelated factors and greater interpretability. The eigenvalue for a given factor reflects the variance in all the variables which is accounted for by that factor. If a factor has a low eigenvalue, then it is contributing little to the explanation of variance in the variables and may be ignored. The factor that had an eigenvalue of more than 1 was considered here. Variables with absolute factor loadings ≥0.50 were considered major contributors to this factor [25]. For further statistical analysis, individual factor scores were saved and divided into quartiles.

To compare the effect of common factors on the risk of carotid atherosclerosis, odds ratios (ORs) and their 95% confidence intervals (CIs) for quartiles (with the first quartile defined as the reference quartile) of each common factor were estimated by unconditional logistic regression models with adjustment for age and education level for both sexes and additional adjustment for smoking status and alcohol drinking for men because of the markedly fewer female smokers (0.7% of female participants were smokers) and female alcohol drinkers (2.0% of female participants were drinkers). Subgroup analysis was conducted according to smoking status in men.

Statistical significance was accepted at a two-sided *P* value of <0.05, and SPSS 16.0 software (SPSS, Inc., Chicago, IL, USA) was used for data analysis.

## Results

### 1. Characteristics of the participants

Of the 3,162 subjects interviewed, 2,755 (772 men and 1983 women) were included in the analysis. The characteristics of the study participants are shown in **Table 1**. The male participants (59.3±5.4 years) were significantly older than the female participants (56.8±4.9 years) (*P*<0.001). Mean carotid IMT was greater in men than in women (CCA: 0.767 vs. 0.688, BIF: 1.003 vs. 0.894, ICA: 0.690 vs. 0.619 mm); the prevalence of carotid atherosclerosis (carotid IMT ≥1.00 mm) was also significantly greater in men than in women (10.5% vs. 3.4% for CCA-IMT, 48.2% vs. 27.1% for BIF-IMT, 4.8% vs. 1.3% for ICA-IMT).

**Table 1:** Characteristics of the study participants by sex.

	Men	Women	*P*-value^a^
	(n = 772)	(n = 1983)	
Age, year	59.3±5.4	56.8±4.9	<0.001
Education level, n (%)			<0.001
Primary school or below	226 (28.9)	608 (30.7)	
High school or secondary school	309 (39.6)	973 (49.0)	
College degree or above	246 (31.5)	402 (20.3)	
Smoking status, n (%)^b^			<0.001
Non-smokers	384 (49.7)	1970 (99.3)	
Smokers	388 (50.3)	13 (0.7)	
Drinking status, n (%)^c^			<0.001
Non-drinkers	650 (84.2)	1944 (98.0)	
Drinkers	122 (15.8)	39 (2.0)	
Anthropometrics			
Body mass index, kg/m^2^	23.8±2.9	23.1±3.2	<0.001
Waist circumference, cm	86.1±8.4	81.1±8.8	<0.001
Waist-to-hip ratio	0.92±0.05	0.87±0.06	<0.001
Blood pressure, mmHg			
Diastolic blood pressure	80.6±10.4	77.4±10.7	<0.001
Systolic blood pressure	126.1±17.0	122.8±17.7	<0.001
Laboratory tests, mmol/L			
Total cholesterol	5.10±1.00	5.55±1.06	<0.001
HDL-C	1.22±0.28	1.44±0.33	<0.001
LDL-C	3.46±0.86	3.68±0.92	<0.001
TG	1.71±1.44	1.53±1.13	0.002
Glu	4.86±1.07	4.71±0.99	<0.001
UA	338.3±107.0	260.8±104.5	<0.001
Carotid IMT, mm^d^			
CCA-IMT	0.767±0.203	0.688±0.139	<0.001
BIF-IMT	1.003±0.263	0.894±0.193	<0.001
ICA-IMT	0.690±0.181	0.619±0.133	<0.001
Carotid atherosclerosis, n (%)^e^			
Increased CCA-IMT	81 (10.5)	68 (3.4)	<0.001
Increased BIF-IMT	372 (48.2)	537 (27.1)	<0.001
Increased ICA-IMT	37 (4.8)	25 (1.3)	<0.001

Abbreviations: HDL-C: high density lipoprotein-cholesterol; LDL-C: low density lipoprotein-cholesterol; TG: total triglycerides; Glu: glucose; UA: uric acid; IMT: intima-media thickness; CCA: common carotid artery; BIF: bifurcation artery; ICA: internal carotid artery.

Data are expressed as mean ± SD unless otherwise indicated.

a Student's *t* test for continuous variables and chi-square test for categorical variables.

b Smoker was defined by having smoked greater than 5 packs during their lifetime and reporting smoked at the time of the interview.

c Drinker was defined by having had wine 1 time(s) weekly for at least six consecutive months and reporting drinked at the time of the interview.

d Carotid CCA, BIF, and ICA IMTs were taken as the mean value of the left and right CCA, BIF, and ICA IMTs.

e Carotid atherosclerosis was defined as a CCA, BIF, and ICA IMT ≥1.00 mm.

Of the 11 selected variables, BMI, WC, WHR, DBP, SBP, TG, Glu, and UA were higher in men than in women, but TC, HDL-C, and LDL-C were lower in men than in women (all *P*<0.01) (**Table 1**). For other factors, men reported higher education levels, and current smoking and alcohol drinking was more prevalent among men than among women (all *P*<0.001) (**Table 1**).

### 2. Identification of common factors

Of the 11 metabolic factors, four common factors were extracted for both men and women that fit the criterion of an eigenvalue >1.0 (**Table 2**). These factors and their order of their importance were similar for men and women. For both men and women, Factor 1 was the most powerful factor comprising mainly positive loadings for WC, WHR, and BMI, and was designated the “adiposity” factor. Factor 2, the “blood lipids” factor, loaded heavily on TC and LDL-C. Factor 3, we labeled “TG/UA” for men and “TG/UA/Glu” for women. TG and UA had high positive loadings in men, and Glu contributed substantially to this factor in women. Factor 4, the “blood pressure” factor, mainly comprised positive SBP and DBP loadings. These four factors cumulatively accounted for 73.8% and 74.9% of the variance in the measured variables in men and women, respectively (**Table 2**).

**Table 2:** Varimax-rotated factor loading scores by sex^a^.

Risk factors	Men (n = 772)				Women (n = 1983)			
	Adiposity	Blood lipids	TG/UA	BP	Adiposity	Blood lipids	TG/UA/Glu	BP
Waist circumference	**0.953**				**0.955**			
Waist-to-hip ratio	**0.884**				**0.840**			
Body mass index	**0.861**				**0.836**			0.206
Total cholesterol		**0.937**	0.245			**0.945**	0.202	
LDL-C		**0.911**				**0.927**		
HDL-C	−0.390	0.493	−0.329		−0.440	0.426	−0.413	
TG			**0.908**				**0.891**	
UA			**0.823**				**0.813**	
Glu			0.331				0.488	
Systolic BP				**0.877**				**0.891**
Diastolic BP				**0.870**				**0.890**
% Total variance	24.3	17.8	16.9	14.7	23.6	17.8	17.9	15.5
% Cumulative variance	24.3	42.2	59.0	73.8	23.6	41.5	59.4	74.9

Abbreviations: BP: blood pressure; HDL-C: high density lipoprotein-cholesterol; LDL-C: low density lipoprotein-cholesterol; TG: total triglycerides; UA: uric acid; Glu: glucose.

a Factors were determined by principal components factor analysis. Factor loading scores with absolute values of ≥0.20 are shown in the table.

### 3. Common factors and carotid atherosclerosis

The predictive power of these four common factors was evaluated by comparing the adjusted ORs of either the upper quartiles (with the first quartile used as the reference) (**Table 3**). In men, the ORs of developing carotid atherosclerosis increased steeply with increases in the “adiposity” factor score and to a lesser extent with increases in the “blood pressure” factor score, but no significant association was observed for the “blood lipids” factor or “TG/UA” factor. The adjusted ORs (95% CIs) comparing the extreme quartiles for increased CCA-IMT were 4.64 (2.04–10.59) for the “adiposity” factor (*P* trend<0.001), 2.85 (1.37–5.90) for the “blood pressure” factor (*P* trend = 0.017), 2.00 (0.94–4.26) for the “TG/UA” factor (*P* trend = 0.879), and 1.26 (0.63–2.50) for the “blood lipids” factor (*P* trend = 0.272). For increased BIF-IMT, the “adiposity” factor remained significantly associated, and the OR (95% CIs) for quintile 4 vs. quintile 1 was 2.37 (1.54–3.64) (*P* trend<0.001). However, no association was observed for increased ICA-IMT (*P* trend: 0.183–0.560).

**Table 3:** Odds ratio (95% CIs) of carotid atherosclerosis for quartiles of the four common factor scores^a^.

	Quartile 1	Quartile 2	Quartile 3	Quartile 4	*P*-trend
**Men (n = 772)**					
Increased CCA-IMT					
Adiposity	1.00	2.44 (1.01–5.90)	3.59 (1.52–8.47)	**4.64 (2.04–10.59)**	**0.000**
Blood pressure	1.00	1.84 (0.86–3.96)	1.61 (0.73–3.56)	**2.85 (1.37–5.90)**	**0.017**
Blood lipids	1.00	1.20 (0.60–2.37)	0.81 (0.39–1.69)	1.26 (0.63–2.50)	0.272
TG/UA	1.00	1.90 (0.87–4.15)	2.32 (1.10–4.94)	2.00 (0.94–4.26)	0.879
Increased BIF-IMT					
Adiposity	1.00	1.18 (0.77–1.81)	1.05 (0.69–1.62)	**2.37 (1.54–3.64)**	**0.000**
Blood pressure	1.00	1.35 (0.88–2.07)	1.33 (0.87–2.04)	1.36 (0.89–2.10)	0.160
Blood lipids	1.00	1.13 (0.74–1.74)	0.96 (0.63–1.48)	1.16 (0.76–1.79)	0.402
TG/UA	1.00	0.66 (0.43–1.02)	1.49 (0.97–2.30)	1.11 (0.72–1.70)	0.097
Increased ICA-IMT					
Adiposity	1.00	0.41 (0.12–1.39)	0.88 (0.31–2.47)	1.66(0.70–3.95)	0.216
Blood pressure	1.00	0.58 (0.20–1.69)	0.45 (0.14–1.42)	1.68(0.70–4.01)	0.183
Blood lipids	1.00	0.84 (0.32–2.24)	0.80 (0.30–2.13)	0.76(0.28–2.07)	0.437
TG/UA	1.00	1.23 (0.41–3.62)	1.72 (0.60–4.92)	2.15(0.79–5.87)	0.560
**Women (n = 1983)**					
Increased CCA-IMT					
Adiposity	1.00	2.37 (0.91–6.12)	2.16 (0.83–5.65)	**2.88 (1.14–7.23)**	**0.028**
Blood pressure	1.00	1.73 (0.75–4.00)	1.65 (0.71–3.81)	2.19 (0.99–4.86)	**0.016**
Blood lipids	1.00	0.87 (0.41–1.81)	0.75 (0.34–1.64)	1.53 (0.79–2.96)	0.435
TG/UA/Glu	1.00	2.19 (0.83–5.77)	1.99 (0.76–5.22)	**3.41 (1.38–8.42)**	**0.010**
Increased BIF-IMT					
Adiposity	1.00	1.22 (0.91–1.66)	1.04 (0.77–1.41)	**1.55 (1.15–2.01)**	**0.004**
Blood pressure	1.00	0.95 (0.70–1.29)	1.25 (0.93–1.68)	**1.72 (1.29–2.30)**	**0.000**
Blood lipids	1.00	1.43 (1.06–1.92)	1.25 (0.93–1.69)	**1.55 (1.16–2.09)**	**0.005**
TG/UA/Glu	1.00	1.17 (0.87–1.59)	1.22 (0.91–1.65)	**1.54 (1.14–2.07)**	**0.002**
Increased ICA-IMT					
Adiposity	1.00	1.56 (0.44–5.52)	0.86 (0.21–3.52)	2.19 (0.65–7.40)	0.128
Blood pressure	1.00	0.13 (0.02–1.08)	1.30 (0.48–3.49)	0.89 (0.30–2.61)	0.861
Blood lipids	1.00	1.96 (0.35–10.87)	5.54 (1.21–25.46)	3.59 (0.75–17.16)	**0.012**
TG/UA/Glu	1.00	0.92 (0.23–3.75)	1.44 (0.41–5.07)	2.18 (0.65–7.27)	0.108

Abbreviations: TG: total triglycerides; UA: uric acid; Glu: glucose; IMT: intima-media thickness; CCA: common carotid artery; BIF: bifurcation artery; ICA: internal carotid artery.

a Odds ratios (95% CI) were estimated by multivariate unconditional logistic regression models. Covariates adjusted: age and educational level for women; + smoking status and alcohol drinking for men.

In women, these four factors contributed in similar way to the presence of carotid atherosclerosis (**Table 3**). In comparison to the first quartile, ORs for the highest quartile ranged from 1.54 to 1.72 for increased BIF-IMT (*P* trend: <0.001–0.005). Significantly increased risks remained for the “adiposity” and “TG/UA/Glu” factors for increased CCA-IMT (*P* trend: 0.028 for the “adiposity” factor; 0.010 for the “TG/UA/Glu” factor) and for the “blood lipids” factor for increased ICA-IMT (*P* trend = 0.012).

Subgroup analysis of men based on smoking status revealed that the “adiposity” factor was still the strongest predictor for the increased CCA-IMT and BIF-IMT (**Table 4**).

**Table 4:** Odds ratios (95% CIs) of carotid atherosclerosis for a 1-SD increase in common factor scores according to smoking status among men^a^.

	Non-smokers (n = 384)		Smokers (n = 388)	
	OR (95% CIs)	*P*-value	OR (95% CIs)	*P*-value
Increased CCA-IMT				
Adiposity	**1.99 (1.29–3.08)**	**0.002**	**1.48 (1.10–2.00)**	**0.011**
Blood pressure	1.37 (0.94–2.01)	0.105	1.32 (0.97–1.79)	0.074
Blood lipids	0.99 (0.67–1.46)	0.958	1.25 (0.92–1.69)	0.160
TG/UA	1.16 (0.71–1.89)	0.560	0.98 (0.69–1.40)	0.910
Increased BIF-IMT				
Adiposity	**1.42 (1.12–1.80)**	**0.003**	**1.30 (1.06–1.59)**	**0.010**
Blood pressure	1.10 (0.88–1.36)	0.404	1.13 (0.91–1.39)	0.154
Blood lipids	1.02 (0.82–1.27)	0.861	1.14 (0.92–1.40)	0.226
TG/UA	1.04 (0.78–1.39)	0.783	1.19 (0.97–1.47)	0.095
Increased ICA-IMT				
Adiposity	1.51 (0.79–2.87)	0.213	1.16 (0.78–1.72)	0.477
Blood pressure	0.96 (0.57–1.82)	0.900	1.41 (0.94–2.10)	0.094
Blood lipids	1.17 (0.62–2.22)	0.636	1.15 (0.76–1.72)	0.512
TG/UA	0.88 (0.35–2.22)	0.790	1.16 (0.80–1.70)	0.437

Abbreviations: TG: total triglycerides; UA: uric acid; IMT: intima-media thickness; CCA: common carotid artery; BIF: bifurcation artery; ICA: internal carotid artery.

a Odds ratios (95% CI) were estimated by multivariate unconditional logistic regression models after adjusted for age, educational level, and alcohol drinking.

## Discussion

In the present study, we identified four risk factors through PCFA for both men and women: “adiposity,” “blood lipids,” “TG/UA” (in men) or “TG/UA/Glu” (in women), and “blood pressure,” which accounted for at least 70% of the total variation in both sexes. Of these common factors, the “adiposity” factor was the strongest predictor of carotid atherosclerosis in men, followed by the “blood pressure” factor; however, these four factors contributed similarly to carotid atherosclerosis in women.

Our analysis showed the “obesity” factor, typified by high factor loadings for BMI, WC, and WHR, to be the strongest independent risk factor for atherosclerosis in men. This finding contrasts with findings of most previously reported epidemiologic studies. Yusuf et al. [1], using data from the INTERHEART study, which involved individuals from 52 high, middle, and low income countries and included 6,086 Chinese, suggested that smoking and elevated apolipoproteins (ApoB/ApoA1 ratio) were the most important risk factors for myocardial infarction across geographic regions and in both sexes, and abdominal obesity, as well as other factors such as psychosocial characteristics, diabetes, and hypertension, were the next most important risk factors in men and women. O'Donnell et al. [26], using data from the INTERSTROKE study involving individuals from 22 middle and low income countries, reported that hypertension was the strongest risk factor for all stroke subtypes in men and women, and a much stronger association between WHR and stroke risk was observed in women than in men. Though not the strongest, findings of both Yusuf et al. [1] and O'Donnell et al. [26] suggested a strong, independent, and graded relation between the obesity indicator and the risks of myocardial infarction and stroke in men.

We found the “blood pressure” factor to be the second strongest predictor of atherosclerosis in men, a finding that was consistent with the findings of a meta-analysis of 45 prospective cohort studies [27] and a cohort study in 5092 Chinese male steelworkers aged 18–74 years [28]. In these studies, blood pressure was shown to be a much stronger predictor of CVD than other factors, e.g. blood cholesterol. In anther cohort of more than 37,000 Chinese, 49.5% of the excess annual mortality from stroke was attributable to hypertension, much higher than that other factors [29]. These findings seem to support the important role of blood pressure in carotid atherosclerosis in our population.

In contrast to Yusuf et al. [1] and O'Donnell et al. [26], we failed to identify any predominating risk factors for atherosclerosis in women. Our data show that these four common factors had an similar adverse effect on IMT in women. A meta-analysis reported that the relative risk of CVD associated with the metabolic syndrome was higher in women than in men [30]. It is possible that differences in study methodologies, criteria used to recruit participants, specific variables analyzed, and sample sizes can explain the sex-based difference in the risk profiles for atherosclerosis [31].

Atherosclerosis is a complex process that might affect very specific sites of the vasculature, and there might be a segment-based difference in the CVD risk factors and the risk of atherosclerosis [32]. We found CCA-IMT to be the most sensitive indicator of atherosclerosis in men and BIF-IMT to be the strongest indicator of CVD risk in women. Findings from the British Regional Heart Study suggested that CCA-IMT was strongly associated with risk factors for stroke, whereas BIF-IMT was more directly associated with risk factors for ischemic heart disease [33]. The lack of association for ICA-BIF is probably due to relative substantial measurement error, leading to greater variation in the association [34], [35].

An important strength of the present study is the use of factor analysis to derive common factors to assess the associations between CHD risk factors and atherosclerosis. As we know, factors such as BMI and cholesterol correlate strongly with each other, and traditional statistical methods such as multiple regression analysis cannot identify and quantify the interrelationships among these risk factors for CHD and may even mask the true associations. Factor analysis is a multivariate analysis technique that can be used to investigate the clustering and interdependence of risk variables [36], [37]. Through factor analysis, we identified similar common factors that were identified in previous studies [13]. Other strengths include the relatively large sample size, which provided enough power and precision in the estimates and in all indices used in the present study for us to conclude that the measures were objective and that the variations were acceptable, thus avoiding recall bias and reducing measurement errors.

Nonetheless, a number of potential limitations should be noted. First, the direction of causality cannot be inferred from cross-sectional studies. We minimized this factor by excluding individuals with any previously confirmed chronic disease such as diabetes, CVD, dyslipidemia, or cancer, which might have substantially altered lifestyles or risk factor levels contributing to progression of atherosclerosis. We also excluded individuals taking any medication known to affect lipids or glycometabolism. Another limitation is that the study group comprised volunteers recruited from communities rather than a population-based random sample and are therefore unlikely to have reflected the prevalence of risk factors in the entire region. However, the key to ensuring internal validity of the study is to compare the relative importance of these factors in our population, which we have done. In addition, the prevalence of cardiovascular risk factors in our study closely matches the data for similar age-groups and sexes from the 2007–2008 China National Diabetes and Metabolic Disorders Study [38]. Thus our overall conclusions should be broadly applicable to the Chinese population.

In conclusion, our study showed the “adiposity” factor and “blood pressure” factor to be the strongest risk factors for carotid atherosclerosis in men, whereas the four common factors contributed equally to the risk of carotid atherosclerosis in women. These findings argue for specific and intensive strategies for the management of adiposity and blood pressure in men and comprehensive prevention strategies in women.
